# Enhanced antitumor and anti-angiogenic effects of metronomic Vinorelbine combined with Endostar on Lewis lung carcinoma

**DOI:** 10.1186/s12885-018-4738-2

**Published:** 2018-10-11

**Authors:** Rong-Sheng Qin, Zhen-Hua Zhang, Neng-Ping Zhu, Fei Chen, Qian Guo, Hao-Wen Hu, Shao-Zhi Fu, Shan-Shan Liu, Yue Chen, Juan Fan, Yun-Wei Han

**Affiliations:** 1 Suining first people’s hospital, Sichuan Province, Suining, 629000 China; 2 Department of Oncology, the Affiliated Hospital of Southwest Medical University, Sichuan Province, Luzhou, 646000 China; 3 Department of Nuclear Medicine, the Affiliated Hospital of Southwest Medical University, Sichuan Province, Luzhou, 646000 China

**Keywords:** Anti-angiogenesis, Endostar, Metronomic chemotherapy, Vinorelbine

## Abstract

**Background:**

Conventional chemotherapy is commonly used to treat non-small cell lung cancer (NSCLC) however it increases therapeutic resistance. In contrast, metronomic chemotherapy (MET) is based on frequent drug administration at lower doses, resulting in inhibition of neovascularization and induction of tumor dormancy. This study aims to evaluate the inhibitory effects, adverse events, and potential mechanisms of MET Vinorelbine (NVB) combined with an angiogenesis inhibitor (Endostar).

**Methods:**

Circulating endothelial progenitor cells (CEPs), apoptosis rate, expression of CD31, vascular endothelial growth factor (VEGF), hypoxia inducible factor-1 (HIF-1α) were determined using flow cytometry, western blot analysis, immunofluorescence staining and Enzyme-linked immunosorbent assay (ELISA) analysis. And some animals were also observed using micro fluorine-18-deoxyglucose PET/computed tomography (^18^F-FDG PET/CT) to identify changes by comparing SUVmax values. In addition, white blood cell (WBC) counts and H&E-stained sections of liver, lungs, kidney, and heart were performed in order to monitor toxicity assessments.

**Results:**

We found that treatment with MET NVB + Endo was most effective in inhibiting tumor growth, decreasing expression of CD31, VEGF, HIF-1α, and CEPs, and reducing side effects, inducing apoptosis, such as expression of Bcl-2, Bax and caspase-3. Administration with a maximum tolerated dose of NVB combined with Endostar (MTD NVB + Endo) demonstrated similar anti-tumor effects, including changes in glucose metabolism with micro fluorine-18-deoxyglucose PET/computed tomography (^18^F-FDG PET/CT) imaging, however angiogenesis was not inhibited. Compared with either agent alone, the combination of drugs resulted in better anti-tumor effects.

**Conclusion:**

These results indicated that MET NVB combined with Endo significantly enhanced anti-tumor and anti-angiogenic responses without overt toxicity in a xenograft model of human lung cancer.

## Background

Lung cancer is one of the most commonly diagnosed malignant tumors and non-small cell lung cancer (NSCLC) accounts for approximately 80–85% of all lung cancer diagnosis. Lung cancer exhibits a high rate of mortality and is often not diagnosed at an early stage. Although conventional chemotherapy has beneficial therapeutic effects, a major limitation of this type of chemotherapy is that the side-effects of the drugs reduce the quality of a patient’s life. Because drug resistance is commonly seen in cases with NSCLC, more effective treatment strategies should be explored.

Metronomic chemotherapy (MET) is defined as a therapeutic approach by chronic administration of chemotherapeutic agents at a relatively low and minimally toxic dose without a prolonged drug-free break [1]. The mechanism involved exerts its anti-tumor effects by anti-angiogenic mechanisms, for example by inducing endothelial cell apoptosis, or by reducing viable circulating endothelial progenitor cells (CEPs) [2]. Previous studies have suggested that MET may be a multi-targeted anti-tumor strategy that restores anti-tumor immunity and induces tumor dormancy. Tumor growth and metastasis by antagonizing angiogenesis are also inhibited [3]. Previous studies have shown that metronomic oral Vinorelbine can be safely used in elderly patients with advanced NSCLC, allowing for long-term disease stabilization combined with optimal patient compliance [4, 5]. Together, these studies, including numerous preclinical and clinical trials, provide accumulative evidence that MET maintains the therapeutic response, minimizes relapse after conventional chemotherapy, and overcomes resistance [1–3].

The inhibitor of angiogenesis, Endostar, is a modified recombinant human endostatin that is derived from rat vascular endothelial tumor cells [6] and inhibits tumor endothelial cell proliferation, angiogenesis, and tumor growth. Antimitotics, including taxanes and vinca alkaloids are lead drugs for metronomic treatment as they inhibit angiogenesis through multiple mechanisms [7]. Previous studies have shown that antitumor drugs can inhibit tumor cell growth effects on different cell cycles, and may induce tumor cell apoptosis. Vinorelbine binds to tubulin, thereby preventing formation of the mitotic spindle, which leads to cell death [8]. Vinorelbine is a semisynthetic vinca alkaloid and, as an oral formulation, is favored in the chronic administration protocol of MET [4, 5, 9]. Given this major advantage and necessity when using oral chemotherapy drugs in the clinic or in preclinical MET treatment approaches [4, 5], Vinorelbine was chosen as the prime candidate in this study. Several studies have reported that anti-angiogenic drugs combined with metronomic chemotherapy is used in for the treatment of advanced NSCLC and being investigated in various types of cancer, including cancer of the prostate and breast [10–12]. However, it is unknown whether Endostar combined with MET NVB enhances anti-tumor and anti-angiogenic effects in advanced stages of NSCLC. We investigated whether Endostar combined with MET NVB or Endostar combined with MTD NVB is superior regarding anti-tumor effects. We hypothesized that Endostar combined with MET NVB enhanced anti-tumor and anti-angiogenic responses without overt toxicity in a mous xenograft model. To test this hypothesis, we employed a xenograft model to evaluate the role of metronomic Vinorelbine and/or Endostar on the growth and angiogenesis of implanted lung tumors. In addition, we evaluated adverse events induced by the different treatment methods. The goal of this preclinical study was to provide a novel scientific approach to guide future clinical work.

## Methods

### Cell culture and chemicals

The murine Lewis lung carcinoma (LLC) cell line was purchased from the cell resource center of Shanghai institute of life sciences, Chinese academy of sciences (from ATCC) and was maintained in RPMI-1640 medium, supplemented with 10% fetal calf serum, and the catalogue number of the cell lines used in this study was ATCC® Number: CRL-1642™. The recombinant human endostatin, Endostar, was provided by Shandong Simcere Medgenn Bio-pharmaceutical Co. Ltd. (Yantai, Shandong, China) and stored at 4 °C until required. The doses and the schedules of administration of reference drugs were based on previous studies [13, 14].

Injectable Vinorelbine solution was supplied by the Southwest Medical University (Luzhou, China). The dose of Vinorelbine was chosen based on what was previously described [15, 16]. According to the dose conversion table for animal and human body weights, in which the Du Bois formula is used to calculate the body surface area (BSA) of the patient (m^2^): 0.007184 × (patient height in cm)^0.725^ × (patient weight in kg)^0.425^, the MTD of Vinorelbine for mice was 10 mg/kg, and the MET dose was the maximum daily dose of 1/10–1/3. Therefore, doses of 1.5, 2, 2.5, 3, and 3.5 mg/kg were chosen for the preliminary experiments. An optimal 3 mg/kg of Vinorelbine was chosen, based on the maximum antitumor effect and anti-angiogenesis effect (data not shown).

### Animals

Female C57BL/6 J mice (3–4 weeks of age) were acclimatized for at least a week under standard conditions of 24 ± 2 °C and 50 ± 10% relative humidity before they were enrolled in the study. All animals were sacrificed by orbital puncture at second days after treatment. The animal protocol used in this study was reviewed and approved by the Institutional Animal Care and Use Committee of the Southwest Medical University (Luzhou, China).

### Mouse xenograft model and treatments

To establish a mouse xenograft model, a total of 1 × 10^6^ LLC cells were resuspended in 0.1 ml phosphate buffered saline (PBS; pH, 7.0) and injected subcutaneously into the back of each animal near the right axilla. When the tumors reached a size of approximately 200 mm^3^, ninety tumor-bearing mice were randomized into six groups (*n* = 15 mice per group) and treated for 14 consecutive days as follows: i) NS group (negative control), ii) Endostar group (10 mg/kg/day), iii) MET NVB group, metronomic Vinorelbine group, (3 mg/kg body weight (bw) of Vinorelbine every other day), i.v.) MET NVB + Endo group, v) maximum tolerated dose (MTD) NVB group, (10 mg/kg body weight (bw) of Vinorelbine i.p. on days 1 and 8); and vi) MTD NVB + Endo group. All compounds were administrated intraperitoneally (i.p) and mimicked the oral metronomic administration. During the treatment period, tumors were measured every other day using calipers. The tumor volumes were calculated using the following formula: tumor volume (cm^3^) = length × width^2^ × 0.5 and a tumor growth curve was plotted based on tumor size. The tumor growth inhibition rate on day 15 after treatment was determined using the following formula:

$$ \mathrm{Inhibition}\ \mathrm{rate}\ \left(\%\right)=\left(1-\mathrm{A}/\mathrm{B}\right)\times 100\%. $$

$$ \mathrm{A}={\mathrm{Volume}}_{\mathrm{Day}1\ \mathrm{experiment}\ \mathrm{group}}-{\mathrm{Volume}}_{\mathrm{Day}15\ \mathrm{experiment}\ \left(\mathrm{group}\right)} $$

$$ \mathrm{B}={\mathrm{Volume}}_{\mathrm{Day}1\ \mathrm{control}\ \mathrm{group}}-{\mathrm{Volume}}_{\mathrm{Day}15\ \mathrm{control}\ \mathrm{group}.} $$

### Flow cytometry

A single cell suspension of 1 × 10^6^ cells/ml was prepared from isolated tumor tissue, and incubated for 15 min in the dark with 5 μl Annexin V-FITC and 5 μl PI. A total of 100 μl of peripheral blood from each mouse was stained with CD133-FITC (1:20), CD34-APC (1:20), and Flk-1-PE (1:20), and incubated for 30 min in the dark. Next, red blood cells were lysed for 15 min and peripheral blood nuclear cells were collected. CD133^+^CD34^+^Flk-1^+^ cells represented the frequency of CEPs [17–20]. The frequency of CEPs and the apoptosis rate were determined by flow cytometry analysis (BD FACS Calibur, San Jose, CA, USA).

### Immunohistochemistry

Tumors were fixed in 10% neutral-buffered formalin solution, embedded in paraffin, and 4 um thick sections were cut for immunohistochemical analysis. Sections were stained with antibodies directed against CD31, VEGF, and HIF-1α (1:100, Bioworld Technology, Louis Park, MN, USA), and were performed according to the manufacturer’s instructions (Bioworld Technology, Louis Park, MN, USA). Images were taken using an optical microscope (Olympus, Tokyo, Japan). Staining intensity was scored by two independent experienced pathologists. Each sample was graded according to intensity and extent of staining. The intensity of staining was scored as 0 (no staining), 1 (weak staining), and 2 (strong staining). The extent of staining was based on the percentage of positive tumor cells: 0 (no staining), 1 (1–25%), 2 (26–50%), 3 (51–75%), and 4 (76–100%). These two scores were added together for a final score. A case was considered negative if the final score was 0 or 1 (−) or 2 or 3 (±), and positive if the score was 4 or 5 (+) or 6 or 7 (++). In most cases, the two examiners provided consistent results. Any inconsistencies were resolved by discussion to achieve a consensus score.

### Western blot analysis

Tumor samples were homogenized, and centrifuged at 12,000 rpm for 15 min at 4 °C. The protein concentration in supernatant was determined using a BCA colorimetric assay (Thermo Scientific Rockford, IL, USA). Approximately 40 μg of the supernatant was resolved by SDS-PAGE analysis and transferred to nitrocellulose membranes. Membranes were blocked for 1 h with 5% nonfat milk in 1 × PBS and incubated overnight at 4 °C with primary antibodies directed against VEGF Receptor 2, HIF-1α**,** Bcl-2, Bax, and caspase-3 (1:1000 dilution, Cell signaling Technology, Boston, MA, USA). Next, membranes were washed three times 10 min with 1 × PBS and incubated with a peroxidase-conjugated secondary antibody (1:3000 dilution, Cell signaling Technology, Boston, MA, USA) for 1 h under shaking at room temperature. After incubation, membranes were washed three times 10 min with 1 × PBS and proteins were visualized using chemiluminescence. GAPDH was used as an internal reference for protein loading. Signals were quantified using ImageQuant 5.0 software (Molecular Dynamics, Sunnyvale, CA, USA).

### Enzyme-linked immunosorbent assay analysis

Roughly 1 ml of peripheral blood from was collected by orbital puncture in Eppendorf tubes and allowed to naturally coagulate for 10~ 20 min at room temperature. After centrifugation (1800 g) for 10 min at 4 °C, the protein in the serum was precipitated and immediately frozen at 80 °C until further analysis. HIF-1α and VEGF levels were determined by an ELISA kit according to the manufacturer’s guidelines (Beijing Cheng Lin biological technology co, LTD, Beijing, China). A total of 10 μl serum sample and 40 μl of the standard solutions were added to the wells, incubated at 37 °C for 30 min, and washed 5 times with diluted detergent solution. Subsequently, the wash solution was removed, and 50 μl of Enzyme labeling reagent was added to each well, followed by incubation at 37 °C for 30 min. Next, wells were washed 5 times and 50 μl of stop solution was added to each well. The absorbance was read at a wavelength of 450 nm and HIF-1α and VEGF concentrations were calculated using a standard curve*.*

### Micro ^18^F-FDG PET/CT imaging

Positron emission tomography (PET) using 18F-FDG to monitor antitumor effects was used to identify changes in the glucose metabolism. To study the reactivity of tumor tissue in the experimental groups, we performed micro PET/CT scans and image analysis the day after termination of treatment, using an Inveon micro PET/CT animal scanner (Siemens, Munich, Germany). Mice were fasted for 12 h, and anesthetized with 1% pentobarbital (5 ml/kg), injected intravenously with 100–200 mCi FDG via the tail vein, and scanned. After roughly 40 min, PET/CT images were acquired and collected for data analysis that was performed by comparing the maximum of standardized uptake value (SUVmax values).

### Evaluation of side effects and histopathological analysis

Possible side effects were indicated by observation of body weight, diarrhea, and behavior. Peripheral blood was collected by orbital puncture using in heparin-coated tubes to prevent coagulation, and analyzed by the blood cell automatically detect analyzer (Mindray, Shenzhen, China). After mice were sacrificed, liver, lungs, kidney, and heart were harvested for hematoxylin and eosin (H&E) staining. H&E-stained sections were visualized by two pathologists in a blinded manner.

### Statistical analysis

Data are expressed as the mean ± standard deviation. The statistical significance of the differences between treatment groups was determined by one-way analysis of variance (ANOVA) and the average number of pairwise comparisons was determined by Tamhane’s T2 test. *P* < 0.05 was considered statistically significant. Statistical analyses were performed using SPSS software version 19.0 (SPSS, Inc., Chicago, IL, USA).

## Results

### MET NVB combined with Endostar inhibits the growth of xenograft tumors in vivo

**Fig. 1:**
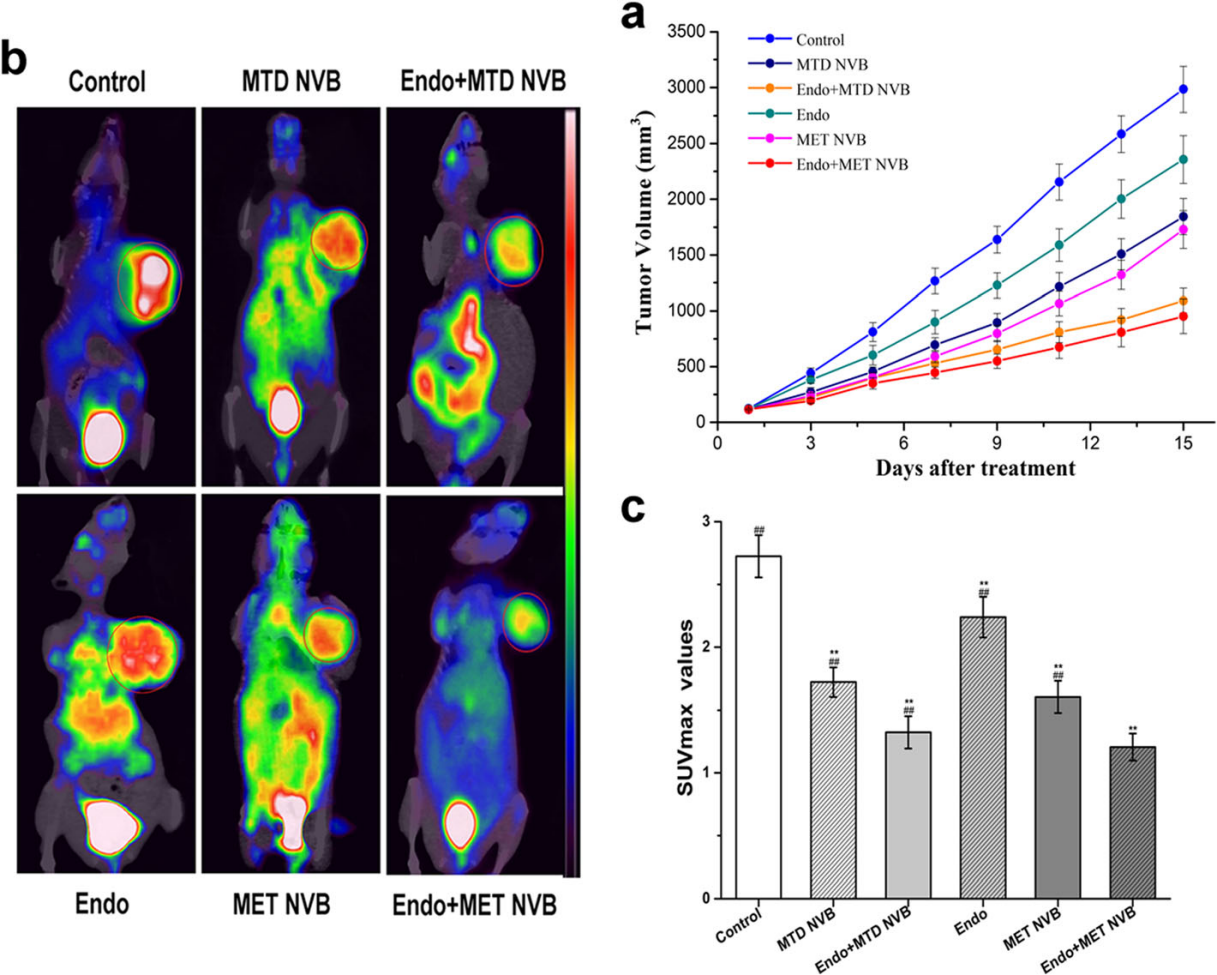
MET NVB treatment combined with Endostar (Endo) inhibits the growth of xenograft tumors in mice. **a** Tumor growth curves in six groups (*n* = 12 for Control, MET NVB, Endo, MET NVB + Endo, MTD NVB, and MTD NVB + Endo groups). Representative ^18^F-FDG PET images (**b**) and SUVmax values (**c**) of mice after one full day of treatment. Data are expressed as the mean ± SD. ^*^P < 0.05, ^**^P < 0.01 versus the control group; ^#^*P* < 0.05, ^##^*P* < 0.01 versus the MET NVB + Endo group

In Fig. 1a, tumor growth curves are presented. The data showed that in the control group the tumors grew rapidly, however, the tumor growth was significantly decreased in all treatment groups (*P* < 0.01). Compared with other groups, the tumor volumes in the MET NVB + Endo group were significantly smaller (*P* < 0.01), except for that of the MTD NVB + Endo group, which indicated that the tumor size was not significantly different between the two groups (*P* > 0.05). Taken together, these data demonstrated that treatment with any drug inhibited the growth of xenograft tumors, and that the MET NVB + Endo group and MTD NVB + Endo group showed the greatest efficacy in tumor growth inhibition compared with other treatment groups (*P* < 0.05 in all cases).

### Micro ^18^F-FDG PET imaging

Representative ^18^F-FDG PET images and SUVmax values of mice in different groups are presented in Fig. 1b, c. The SUVmax value in the MET NVB + Endo treatment group was the lowest when compared to the SUVmax values of the other groups (*P* < 0.05). However, this difference was not significant when compared with the MTD NVB + Endo treatment group (*P* > 0.05). Additionally, both Vinorelbine alone groups and the Endo treatment group showed a decrease in SUVmax values compared to the control group (*P* < 0.05), however the difference between these three groups was not statistically significant. Taken together, these results indicated that the MET NVB + Endo treatment group had a similar effect as the MTD NVB + Endo group, and had an increased tumor growth inhibition effect compared with the other treatment groups tested.

### MET NVB combined with Endostar decreases the frequency of peripheral blood CEPs

**Fig. 2:**
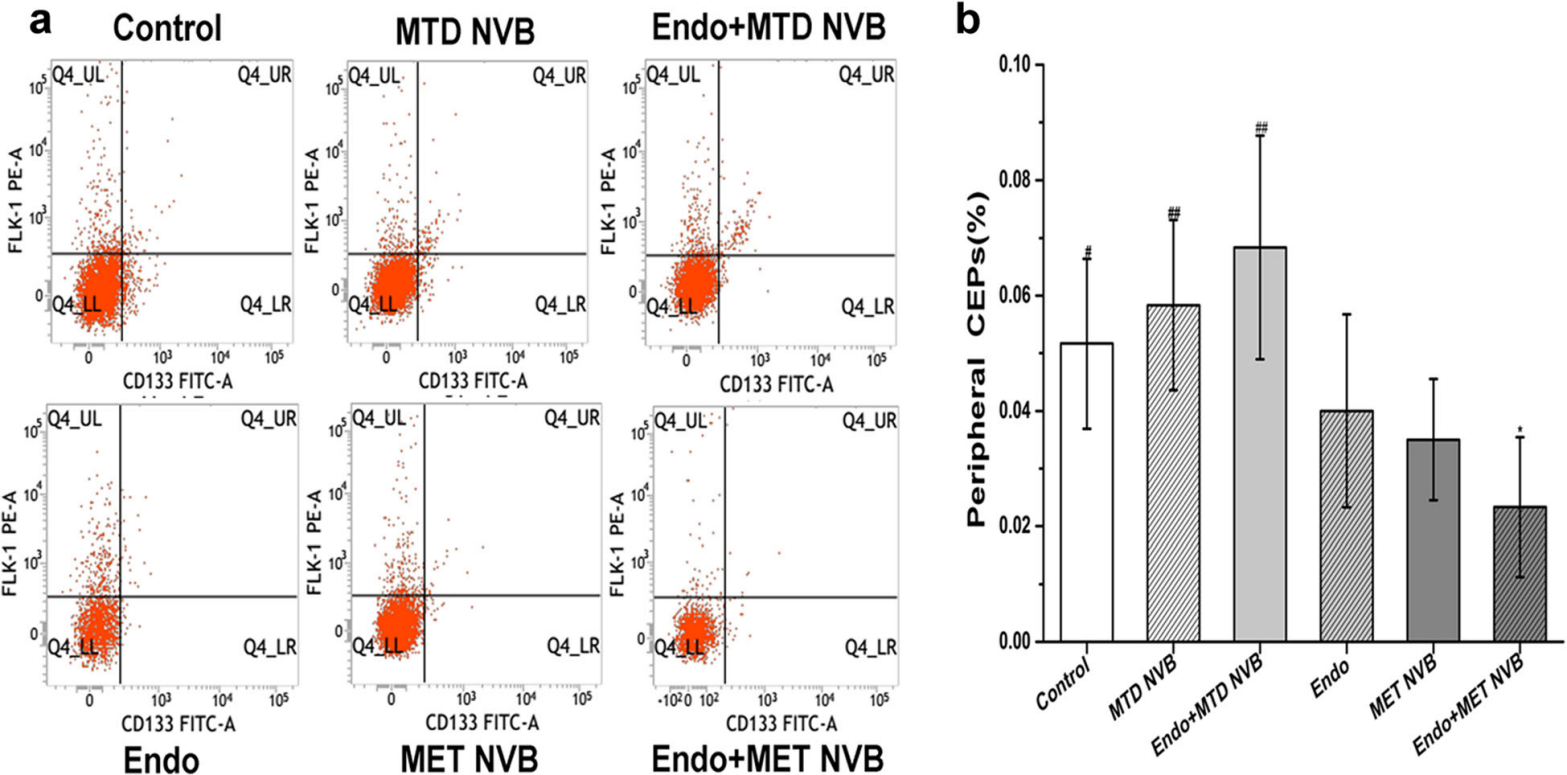
MET NVB treatment combined with Endostar (Endo) decreases the frequency of peripheral blood CEPs. **a** The frequency of peripheral blood CEPs in different groups was determined by flow cytometry analysis. **b** Histogram showing the quantitative data of the mean frequency of CEPs per treatment group. Data are expressed as the mean ± SD. ^*^P < 0.05, ^**^P < 0.01 versus the control group; ^#^*P* < 0.05, ^##^*P* < 0.01 versus the MET NVB + Endo group

We initially characterized the frequency of peripheral blood CEPs in the different treatment groups (Fig. 2a, b). Because the level of CEPs was low, no significant difference was observed in the total number of blood nuclear cells between groups. However, flow cytometry results showed that a significant different in the proportion of CEPs was found when comparing the MET NVB + Endo group with other groups (*P* < 0.05). In particular, the frequency of CEPs in the MET NVB + Endo group (0.023 ± 0.012%) was significantly lower compared to that of all other groups (*P* < 0.05). The frequency of CEPs in the MET NVB group and the Endo group were 0.035 ± 0.01% and 0.04 ± 0.016%. Secondly, a relatively high proportion of peripheral blood CEPs was discovered in the ctrl group, the MTD NVB group, and the MTD NVB + Endo group. Interestingly, treatment of mice with MTD NVB or MTD NVB + Endo increased the frequency of total CEPs (0.058 ± 0.014% or 0.068 ± 0.019%, respectively, *P* < 0.01). Collectively, these data indicated that MET NVB or Endostar significantly decreased the frequency of peripheral blood CEPs, and that combined treatment of both further reduced this frequency in mice.

### MET NVB combined with Endostar reduces tumor-associated microvessel density

**Fig. 3:**
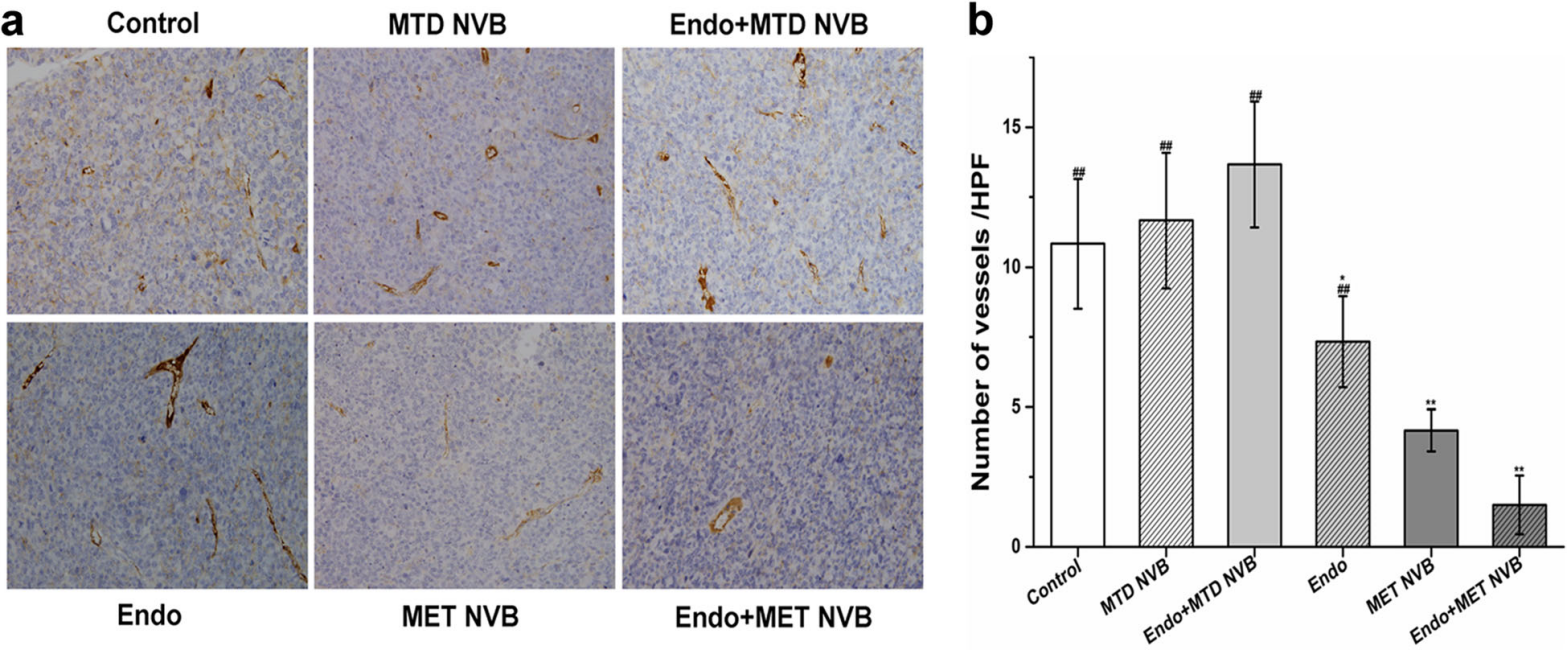
**a** Immunohistochemical images showing CD31 expression changes in tumor tissue in different treatment groups. **b** Quantitative analysis of CD31 expression in tumor tissue of xenograft mice in different groups. Data are expressed as the mean ± SD. ^*^P < 0.05, ^**^P < 0.01 versus the control group; ^#^*P* < 0.05, ^##^*P* < 0.01 versus the MET NVB + Endo group. Original magnification, × 400

As shown in Fig. 3a, immunohistochemical images showing CD31 expression changes in tumor tissue in different treatment groups showed that a high level of MVD was found in the MTD NVB + Endo group, MTD NVB group, and ctrl group, whereas a lower MVD value was observed in the MET NVB group, Endo group, and MET NVB + Endo group. As shown in Fig. 3b, quantitative analysis of CD31 expression in tumor tissue indicated that MVDs from the MET NVB group (4.17 ± 0.75) were different from that of the Endo-treated group of mice (7.33 ± 1.63, *P* < 0.05), and both were greater than that in the MET NVB + Endo group (1.50 ± 1.05, P < 0.05). A relatively high level of CD31 expression in tumor tissue was discovered in the ctrl group (10.83 ± 2.32), the MTD NVB group (11.67 ± 2.42), and the MTD NVB + Endo group (13.67 ± 2.25). Furthermore, differences between the MET NVB + Endo group of mice were particularly notable when compared with all other groups (*P* < 0.01 vs. other groups). Therefore, these data suggested that MET NVB or Endostar, significantly inhibited the formation of microvascular vessels in xenograft tumors.

### MET NVB combined with Endostar decreases expression of VEGF and HIF-1α

**Fig. 4:**
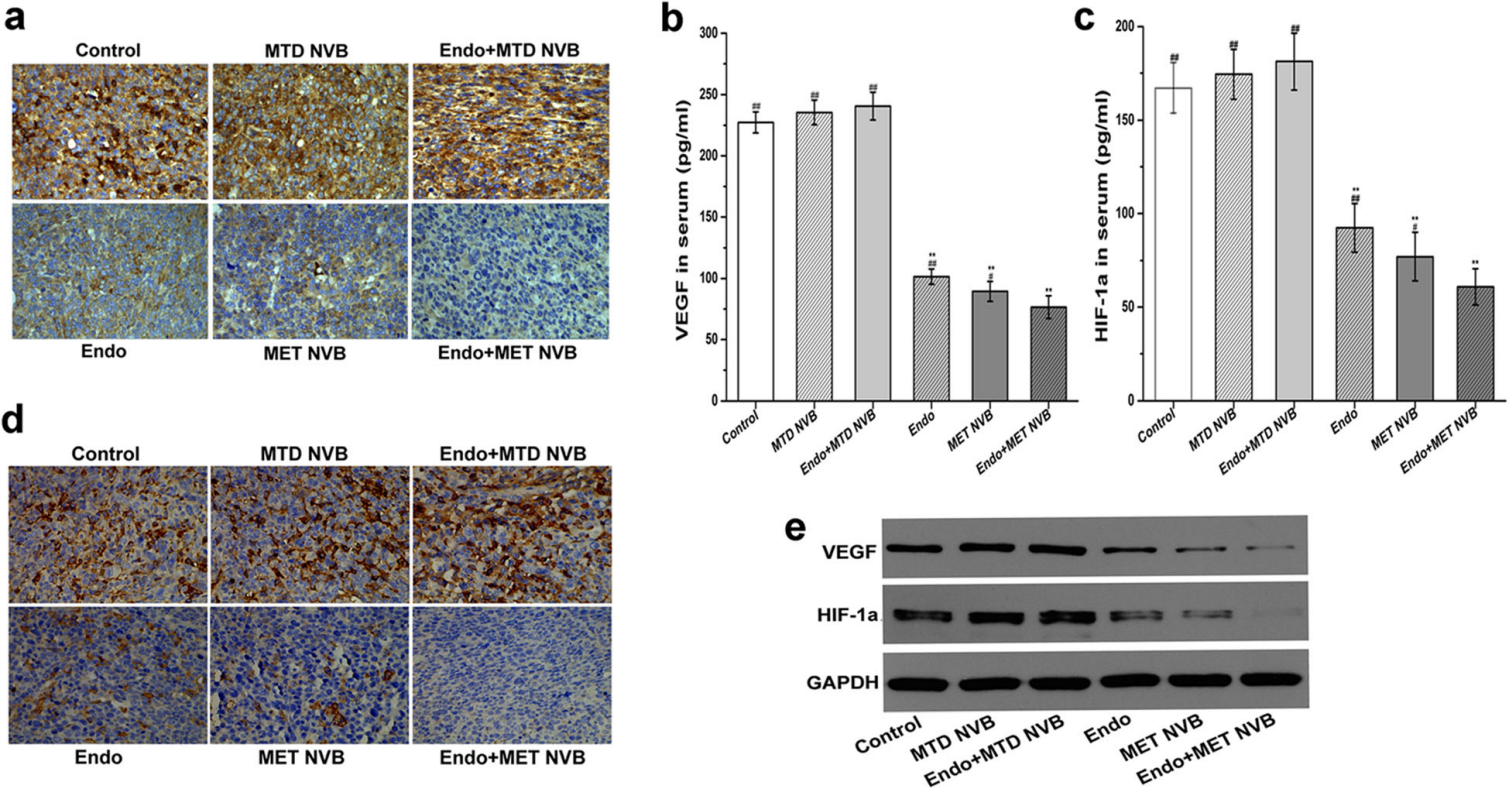
**a** Immunohistochemical analysis showing expression of VEGF in different treatment groups. **b**, **c** Serum levels of VEGF and HIF-1α determined by ELISA assay in different treatment groups. **d** Representative images of immunohistochemical analysis showing expression of HIF-1α in different treatment groups. **e** Expression of VEGF and HIF-1α was determined by Western blot analysis. GAPDH served as the loading control. Data are presented as the mean ± SD. ^*^P < 0.05, ^**^P < 0.01 versus the control group; ^#^*P* < 0.05, ^##^*P* < 0.01 versus the MET NVB+ Endo group

To further verify our findings, ELISA analysis was performed. As shown in Fig. 4b, in the MET NVB + Endo group the serum level of VEGF was 76.52 ± 9.25 pg/ml (P < 0.01 vs. other groups), whereas in the ctrl group showed serum VEGF levels of 227.3 ± 8.55 pg/ml. Moreover, in the MTD NVB + Endo group, the VEGF serum level was 240.54 ± 11.29 pg/ml. Consistent with the results obtained by the ELISA assay, the expression of VEGF and HIF-1α as determined by immunohistochemical analyses were shown in Fig. 4a and d. Compared with the Ctrl group, the expression of VEGF were reduced in the MET NVB group and Endo group, further significantly reduced in the MET NVB + Endo group. However, higher expression levels of VEGF and HIF-1α were observed in the MTD NVB and MTD NVB + Endo group. As shown in Fig. 4e and Table 1, VEGF protein levels as determined using western blot analysis were decreased in the MET NVB + Endo group, but increased in MTD NVB + Endo group. We observed a similar trend of changes for the expression of HIF-1α. In conclusion, we found a reduced expression of VEGF and HIF-1α in the MET NVB + Endo group and a higher expression of VEGF and HIF-1α in the MTD NVB + Endo group.

**Table 1:** Expression of VEGF and HIF-1α in each group

Groups	VEGF/GAPDH	HIF-1α/GAPDH
Control	0.515 ± 0.002 ^**##**^	0.555 ± 0.002 ^**##**^
MTD NVB	0.547 ± 0.003	0.754 ± 0.003
Endo+MTD NVB	0.639 ± 0.004 ^**##**^	0.868 ± 0.004 ^**##**^
Endo	0.346 ± 0.001	0.382 ± 0.002
MET NVB	0.181 ± 0.001	0.201 ± 0.001
Endo+MET NVB	0.078 ± 0.001^**^	0.041 ± 0.001^**^

Data are presented as the mean ± SD. ^*^P < 0.05, ^**^P < 0.01 versus the control group; ^#^*P* < 0.05, ^##^*P* < 0.01 versus the MET NVB+ Endo group

### MET NVB combined with Endostar increases the apoptosis rate of tumor tissue

**Fig. 5:**
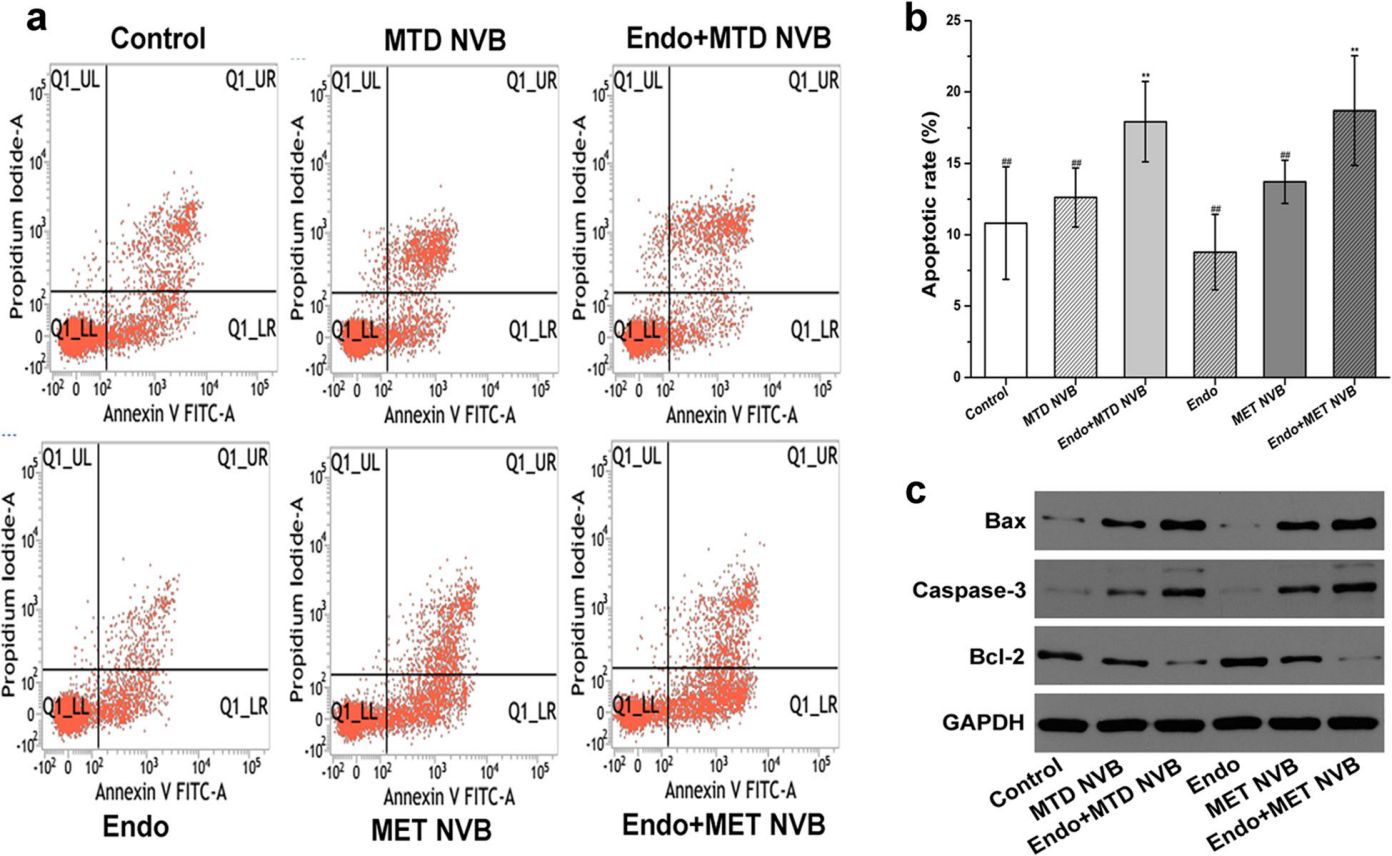
Detection of apoptosis in a Lewis lung carcinoma model. **a** Apoptosis rates of tumor tissue in different treatment groups were performed by flow cytometry analysis. **b** Representative histogram showing significant differences of apoptosis rates in different treatment groups. **c** Expression of Bcl-2, bax, and caspase-3 were determined by Western blot analysis. GAPDH served as the loading control. Data are presented as the mean ± SD. ^*^P < 0.05, ^**^P < 0.01 versus the control group; ^#^*P* < 0.05, ^##^*P* < 0.01 versus the MET NVB+ Endo group

The flow cytometry analyses presented in Fig. 5a, b show that apoptosis rates of the chemotherapy alone treatment groups was very low compared with that of combined treatment groups (*P* < 0.05). Moreover, the apoptosis rate of tumor tissue was significantly increased (*P* < 0.05) after treatment with MET NVB + Endo and MTD NVB + Endo (*P* > 0.05), indicating that treatment with MET NVB + Endo and MTD NVB + Endo had similar effects. Surprisingly, a higher apoptosis rate of tumor tissue was observed in the ctrl group. To assess the effect of the apoptosis rate, we further determined the expression of Bcl-2, Bax, and caspase-3 by Western blot analysis (Fig. 5c and Table 2), which indicated that the amount of Bax and caspase-3 protein was increased, but the Bcl-2 protein level was decreased in the MET NVB + Endo group and MTD NVB + Endo group. Taken together, quantitative analyses indicated that treatment with MET NVB + Endo and MTD NVB + Endo significantly induced apoptosis and caused a synergistic effect.

**Table 2:** Apoptosis rates of tumor tissue in different treatment groups

Groups	Bax/GAPDH	Caspase-3/GAPDH	Bcl-2/GAPDH
Control	0.105 ± 0.001	0.055 ± 0.002	0.529 ± 0.002
MTD NVB	0.443 ± 0.002	0.168 ± 0.001	0.380 ± 0.001
Endo+MTD NVB	0.698 ± 0.003^**^	0.514 ± 0.002^**^	0.148 ± 0.001^**^
Endo	0.068 ± 0.001	0.036 ± 0.001	0.694 ± 0.003
MET NVB	0.571 ± 0.002	0.359 ± 0.001	0.375 ± 0.001
Endo+MET NVB	0.748 ± 0.003^**^	0.602 ± 0.003^**^	0.061 ± 0.001^**^

Data are presented as the mean ± SD. ^*^P < 0.05, ^**^P < 0.01 versus the control group; ^#^*P* < 0.05, ^##^*P* < 0.01 versus the MET NVB+ Endo group

### Toxicity assessments

**Fig. 6:**
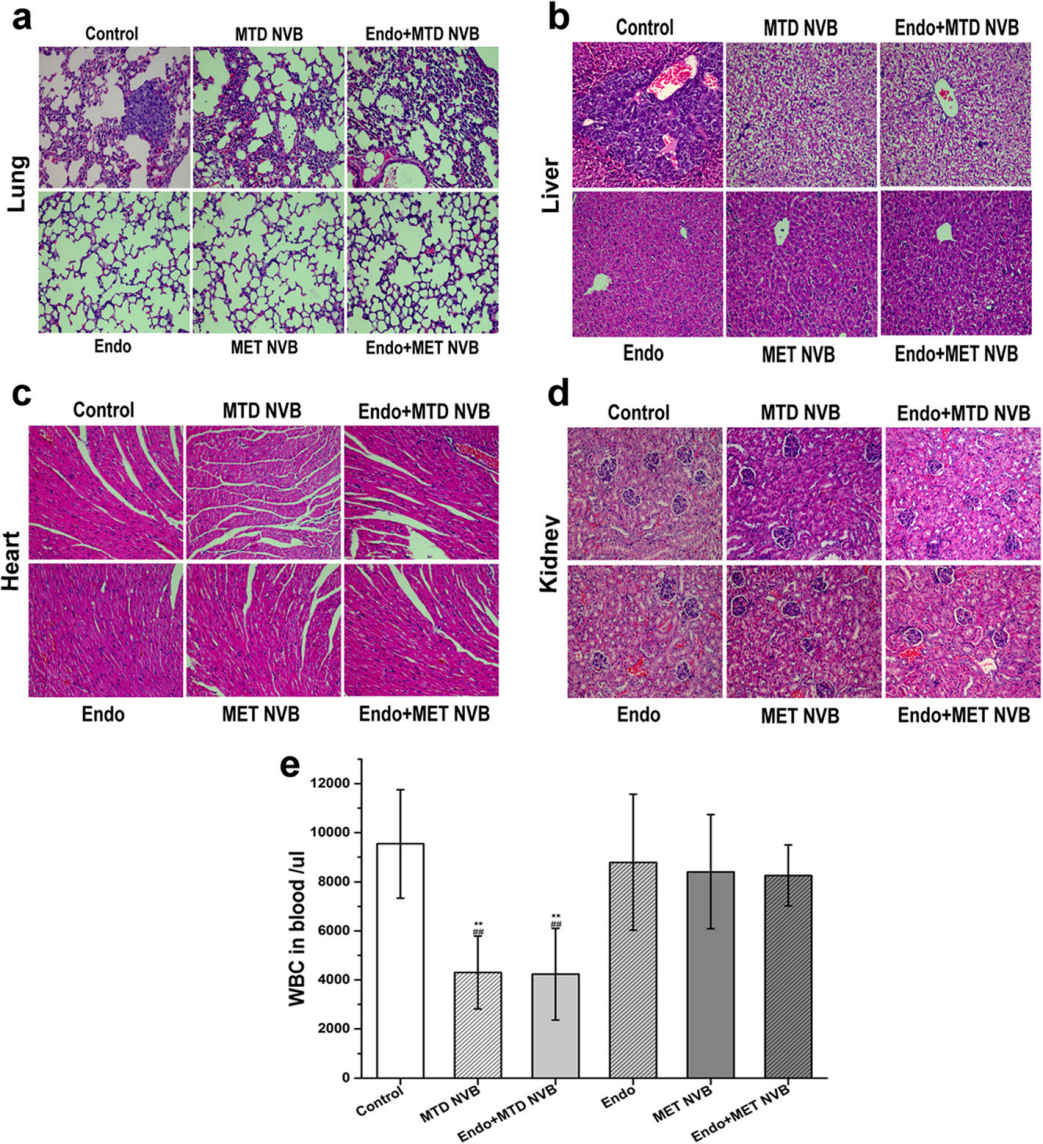
Evaluation of side effects. **a** H&E-stained lung sections of each treatment group (Original magnification, 200). **b** H&E-stained liver sections of each treatment group (Original magnification, 200). **c** H&E-stained heart sections of each treatment group (Original magnification, 200). **d** H&E-stained kidney sections of each treatment group (Original magnification, 200). e Treatment effects on white blood cell (WBC) counts after one full day of treatment. Data are presented as the mean ± SD. ^*^P < 0.05, ^**^P < 0.01 versus the control group; ^#^*P* < 0.05, ^##^*P* < 0.01 versus the MET NVB+ Endo group

To investigate the side effects induced by the different therapeutic regimens, we collected liver, lungs, kidney, and heart tissue and blood to analyze changes in white blood cell (WBC) counts. As shown in Fig. 6, in MTD NVB or MTD NVB + Endo groups WBC counts were (4.3 ± 1.48) × 10^3^ number/μl, or (4.23 ± 1.86) × 10^3^ number/μl (respectively). However, in ctrl group, the WBC count was (9.55 ± 2.2) × 10^3^ number/μl, and in the MET NVB or MET NVB + Endo group, the WBC counts were (8.41 ± 2.32) × 10^3^ number/μl, (8.26 ± 1.23) × 10^3^ number/μl (respectively, *P* > 0.05). These results showed that WBC counts were reduced in the MTD NVB group, while in the MET NVB group WBC counts were within the normal range. H&E-stained sections of the liver, lungs, kidney, and heart of each group were visualized using a light microscope. The H&E-stained sections of the MTD NVB group showed chronic inflammation and interstitial thickening of lung tissue, and hepatic cell edema, degeneration, necrosis and hepatic structural disorders in liver tissue. This was not found it sections derived from the MET NVB group. H&E-stained sections showing macroscopic metastasis of lung and liver tissue were only found in the ctrl group. No organizational changes were found in kidney and heart tissue, and no differences were found between groups.

## Discussion

Advanced stage tumors are not effectively eradicated by conventional chemotherapy because of suboptimal drug targeting, the onset of therapeutic resistance, side effects, and neoangiogenesis. Therefore, novel strategies are required for chemosensitization of cancer cells. MET refers to the close, regular administration of conventional chemotherapy drugs at relatively low, minimally toxic doses with no prolonged break periods [21]. MET is thought to primarily cause antitumor effects by anti-angiogenic activities, both locally by targeting endothelial cells of the tumor neovasculature, and systemically by acting on bone marrow-derived cells, including CEPs [21, 22]. Previous studies have shown that after giving a tumor-burdened mouse a vascular breaker, CEPs can rapidly be mobilized and participate in the regeneration of tumor-associated blood vessels [23, 24]. In a phase III clinical trial of endostatin (ES) in China, the combination of ES and chemotherapy significantly improved the overall and progression-free survival of patients with advanced NSCLC [25]. The combined drug delivery approach led us to hypothesize that metronomic administration of joint anti-angiogenesis drugs may conceptually produce a synergistic antitumor and anti-angiogenesis effect without overt toxicity, and serve as a promising treatment strategy.

Hypoxia inducible factor 1 (HIF-1) is a hypoxia-regulated transcription factor that modulates the expression of numerous hypoxia-inducible genes [26, 27]. The regulatory activity of HIF-1 is determined by the stability of the HIF-1α protein, which is stabilized by hypoxia through an O_2_-dependent degradation domain that rapidly accumulates following exposure to hypoxic conditions [28, 29]. Moreover, hypoxic conditions upregulate the expression of VEGF, thereby promoting tissue permeability and inducing angiogenesis [30, 31]. In tumors, VEGF is the most important angiogenic factor, and inhibits tumor cell apoptosis by inducing the anti-apoptotic protein Bcl-2 [32]. Bcl-2 is an anti-apoptotic protein, and an important modulator of drug-induced apoptosis and chemoresistance [33]. Bax, a pro-apoptotic protein, is found in the cytosol in an inactive form. Caspase-3 and caspase-7 activation are downstream of many proapoptotic signaling pathways [34]. Under anaerobic conditions, CEPs can rapidly be mobilized and participate in the regeneration of tumor blood vessels. CEPs impact intratumoral blood flow and suppress tumor growth by downregulating Bcl-2 and upregulating expression of bax and caspase 3/7.

Vinorelbine is as an oral formulation and that is convenient to be taken. Previous studies have shown that metronomic oral vinorelbine alone can be safely used in elderly patients with advanced NSCLC [4, 5]. But in the present study, we investigated the impact of MET NVB and/or Endostar on the frequency of CEPs, expression of CD31, VEGF, and HIF-1α in tumor-bearing mice. We found that in ctrl-treated mice, peripheral blood CEPs constituted ~ 0.05% of circulating blood cells, which resulted in a higher MVD in xenograft tumors. In contrast, treatment of MET NVB + Endo markedly reduced the frequency of CEPs, MVD, and the expression of VEGF and HIF-1α. Combining drugs also induced better anti-angiogenic responses compared to monotherapy treatment with either drug. Although we found an increase in the frequency of CEPs, expression of CD31, VEGF and HIF-1α in both the MTD NVB and MTD NVB + Endo groups, the differences were not significant. A possible explanation for these findings may be that in cases of high-dose chemotherapy gauge, tumor tissue metabolism and tumor cells are in conditions of hypoxia or insufficient nutrition, and tumor cells promote tumor growth by autophagy degradation and recycling nutrients inside the cell. Anti-angiogenesis therapy may increase tumor hypoxia by promoting tumor cell autophagy and enhancing tumor cell survival ability to cause anti-angiogenesis therapy drug resistance, and vascular formation is promoted by negative feedback regulation. As expected, these findings provide experimental evidence for a synergistic anti-angiogenesis effect of MET and ES, as has recently been shown in a primary tumor model [35].

We also demonstrated that treatment with MTD NVB or/with Endostar effectively inhibited tumor growth and induced apoptosis, but increased tumor vascularity, which is consistent with a role of MTD cytotoxic chemotherapy in inducing endothelial damage [36]. The combination of MET NVB and Endostar significantly enhanced antitumor activity compared to the drugs administered alone, and induced apoptosis by downregulating Bcl-2 expression and upregulating expression of bax and caspase 3/7. In tumor tissue in the control group, a higher apoptosis rate was observed. The reason for this may be that exponential cellular proliferation and an inefficient vascular supply leads to increased formation of necrosis in the tumor tissue. Another phenomenon that is often found in solid tumors is that an inadequate oxygen supply results in hypoxic conditions within the tumor. Recent studies have shown that a combination drug administration approach produces no significant synergistic antitumor effects, and that its mechanism of action involves ischemic conditions in solid tumors that can lead to genetic instability and subsequent tumor progression [37, 38]. In contrast, several studies have demonstrated that the administration of combination drugs had improved efficacy compared with drugs that were administered alone. In this regard, several other studies have recently reported circumstances where MET, when using drugs such as cyclophosphamide, can induce vessel normalization, increase perfusion, and transiently decrease the level of tumor hypoxia [39–41]. Moreover, several cytokines, adhesion molecules, and the internal environment caused a corresponding change to achieve the desired antitumor effect by inhibiting the formation of new blood vessels. The results presented in our study were consistent with these findings [35].

Previous studies have suggested that tumor growth and metastasis are inhibited though drugs administered ‘metronomically’ [3, 21]. However, we investigated whether treatment with Endostar combined with MET NVB or administration of Endostar combined with MTD NVB is superior in regarding antitumor effect. An interesting and unexpected finding was the similar antitumor effect in both treatments. This may be a promising and better approach for the treatment of human cancers. The increased level of CEPs may contribute to the repair of the damaged vasculature after MTD chemotherapy (or plus Endostar) and the decreased level of CEPs suppress the repair and recovery of the tumor vasculature, which is indispensable to tumor growth and metastasis. Regarding the underlying mechanism of action for the opposite effects of MTD NVB or MTD NVB + Endo and MET NVB on the level of CEPs, a possible explanation may be their opposite effects on the mobilization of CEPs. Mice treated with MTD NVB experienced a robust CEPs mobilization a few days after the end of drug administration, whereas the numbers of CEPs in mice treated with MET NVB were sustained at a very low level for a prolonged period [42, 43].

Previous studies have shown that metronomic topotecan administrated for two weeks compared to the maximum tolerated dose of topotecan enhanced anti-angiogenic responses and had low toxicity used in a xenograft model of retinoblastoma treatment [44]. In our study, these results also indicated that metronomic Vinorelbine combined with Endo administrated for 14 consecutive days had similar results. Cumulative toxicity over a longer period of time may be supplemented in subsequent experiments, but recent toxicity has been observed in this experiment. WBC counts were reduced in the groups administrated MTD NVB, whereas WBC counts were in the normal range in mice administrated MET NVB. Moreover, H&E-stained sections of the MTD NVB group showed chronic inflammation and interstitial thickening in lung tissue, and hepatic cell edema, degeneration, necrosis, and hepatic structural disorders in liver tissue. These results suggested that Vinorelbine could be an example, which, when given metronomically was not only minimally or non-toxic, but also had little effect when combined with anti-angiogenic agents, and were well compatible with these findings [35].

In the present study, we demonstrated that treatment with MET combined with anti-angiogenesis drugs resulted in robust antitumor effects through enhanced inhibition of tumor-associated angiogenesis, which was consistent with previous findings [45]. It is conceivable that this therapeutic approach can be moved from bench to bedside, particularly for a maintenance therapy in elderly patients with advanced NSCLC to achieve a sustainable tumor control. These patients do not well tolerate side effects, and may benefit by this treatment from a higher quality of life and a longer progression-free survival [46]. However, several studies have reported that anti-angiogenic drugs combined with chemotherapy may exhibit optimal efficacy when administered successively, and that only a short ‘time window’ for optimal results may exist [47, 48]. Therefore, further research to address the optimal combination and administration regime of anti-angiogenic and antitumor drugs, whether it may be simultaneous or sequential, is warranted. In addition, the optimal administration plan and suitable treatment doses and frequency of NVB must be determined in further studies and clinical trials.

## Conclusions

In conclusion, this study reports preclinical proof-of-principle experiments establishing a rationale for the combination of Endo therapy with MET MVB. In our study, decreasing the expression of CD31, VEGF, and HIF-1α, and peripheral blood CEPs, together with inducing apoptosis and reducing side effects, correlated with the tumor microenvironment and the therapeutic responses to angiogenesis inhibitors, which are promising for uncovering the mechanism of action of anti-angiogenic drugs. Our results emphasized the fact that NVB drugs administered ‘metronomically’ combined with Endostar resulted in enhanced antitumor and anti-angiogenic effects without overt toxicity in a xenograft model of human lung cancer, and that effects were similar as NVB drugs administered ‘standardly’ combined with Endostar. These findings may aid in the design of clinical studies to investigate the efficacy and reduced adverse effects of MET combined with an angiogenesis inhibitor for patients with advanced NSCLC, and may serve as an attractive therapeutic modality.

## Abbreviations

^18^F-FDG PET/CT: fluorine-18-deoxyglucose PET/computed tomography
CEPs: circulating endothelial progenitor cells
HIF-1: Hypoxia inducible factor-1
HPF: high power field
MET: Metronomic chemotherapy
MTD: Maximum tolerated dose
MVD: Microvascular density
NSCLC: non-small-cell lung cancer
SUVmax: the maximum of standardized uptake value
VEGF: Vascular epidermal growth factor
